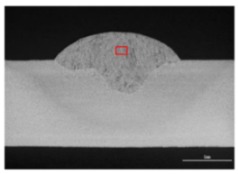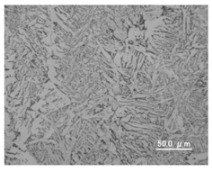# Influence of Current Feeding Position of Duplex Current Feeding MIG Welding on Droplet Heat Quantity

**DOI:** 10.3390/ma12213590

**Published:** 2019-10-31

**Authors:** Atsuhito Aoki, Shinichi Tashiro, Hideaki Kurokawa, Manabu Tanaka

**Affiliations:** 1 Kawasaki Technology Co., Ltd., Akashi 6738666, Japan; aoki_atsu@khi.co.jp; 2 Joining and Welding Research Institute, Osaka University, Osaka 5670047, Japan; tanaka@jwri.osaka-u.ac.jp; 3 Kawasaki Heavy Industries, Ltd., Akashi 6738666, Japan; kurokawa_h@khi.co.jp

**Keywords:** pure argon MIG welding, duplex current feeding, heat quantity of droplet, current feeding points, wettability

## Abstract

Pure argon metal inert gas (MIG) welding is expected to offer the possibility to obtain high toughness weld joints. However, due to its arc instability and low wettability, it is difficult to apply pure argon MIG to a practical welding structure. In order to solve these problems, an improved MIG welding process with a duplex current feeding (DCF-MIG) mechanism was developed. In the DCF-MIG process, the welding current and the wire feeding speed are independently controlled by an additionally feeding secondary current from a secondary power source. Thereby, DCF-MIG can supply a large current compared to conventional MIG under the same deposition rate. In this study, to consider the influence of the secondary current feeding position of DCF-MIG on droplet heat quantity, droplet heat quantity was measured by calorimetry. As a result, the droplet heat quantity was found to be increased significantly with the increase of the distance between the primary current feeding point and secondary current feeding point. The increase of the droplet heat quantity in the DCF-MIG process had a strong effect on improving bead shape and penetration. The droplet heat quantity with the effective current value of DCF-MIG was derived from the simplified calculation and the results roughly agreed with the experimental data.

## 1. Introduction

Both the strength and toughness of weld joints must be ensured for the safety of steel structures used at low temperature, such as Liquefied Natural Gas (LNG) tanks [1,2]. For this purpose, tungsten inert gas (TIG) welding, which is able to obtain stable welding using pure inert shielding gas, is mainly used. Although TIG welding can obtain a high-quality weld joint, it has drawbacks, such as low efficiency. On the other hand, metal inert gas (MIG) welding is a highly efficiency process, but the process tends to be unstable in pure inert shielding gas. To solve this instability problem, inert shielding gas mixed with small amount of oxygen or carbon dioxide is generally used. However, the toughness of weld metal decreases in this case [3,4,5]. Therefore, it is required to develop a stable arc MIG welding process with pure inert shielding gas to achieve both high quality of weld joints and high efficiency. This new MIG process is called “Clean MIG”.

Clean MIG is known to have two problems: One problem is the instability of the arc, which is caused by the unstable behavior of cathode spots, and causes a meandering bead [6,7,8]. Another problem is the formation of the convex bead due to the high surface tension of molten metal [9,10]. Concerning the former problem, various solutions have been reported. For example, Uezono et al. installed the digital filtering process to improve the stability of the weld power source. This filtering process ignores the abnormal high voltage linked with the cathode spot wandering. This method can reduce the effect of the disturbance of feedback voltage [11]. Nakamura et al. developed a coaxial hybrid wire composed of an outer shell with a high melting point and a core metal with a low melting point. During welding, the core metal melts earlier than the outer shell, which makes the liquid column short and reduces the drift of the arc [6]. In an experiment of this coaxial hybrid wire method applied to a similar metal 9%Ni steel welding, as a result, the tensile strength of the weld joint was 855 MPa, the proof stress was 710 MPa and the toughness at 77 K was 89 J. These properties are equal to those of a TIG welding joint. Zenitani et al. introduced a process which used the shielding gas added minimum amount of oxygen necessary to stabilize the arc. This shielding gas is supplied from the vicinity of the contact tip. Consequently, the arc was stabilized and oxygen content in the weld metal was minimized to less than 100 ppm [8]. Kanemaru et al. have examined the optimum welding parameters of the TIG-MIG hybrid welding process. TIG-MIG process was found to make the MIG arc stable despite the use of pure argon shielding gas. It was suggested that arc stabilization by the TIG-MIG process was caused by an increase of thermionic emission by TIG heating and repulsion of the TIG arc and the MIG arc by electromagnetic force [12]. Tanaka et al. reported that the plasma MIG welding process has the ability to stabilize the MIG arc. The plasma MIG enables the ionization of the shielding gas flow in the upstream region of the arc. This flow can stabilize droplet transfer and keep the liquid column at the tip of the welding wire stable by inward electromagnetic force. As a result, the MIG arc was shown to be sufficiently stabilized in pure argon shielding gas [13].

The studies presented improved the arc stability in the Clean MIG process. However, the latter problem of Clean MIG, i.e., convex weld bead, has not yet been solved. A convex weld bead is caused by a high surface tension due to low oxygen content in the molten metal. The oxygen in the molten metal is effective in reducing surface tension, however, in Clean MIG, this effect is not expected. Therefore, one solution is to increase the heat input to the molten metal to reduce the surface tension. Hence, it is desirable to raise the heat quantity of droplets to improve the convex bead. However, due to the unique relationship between the welding current and the wire feed speed in the MIG welding process, it is difficult to increase the heat quantity of droplets independently.

To realize Clean MIG process, in order to obtain both arc stability and a good bead shape, a MIG welding process with a duplex current feeding (DCF-MIG) has been developed [14,15,16,17]. The DCF-MIG welding process can control the welding current and the wire feeding speed independently by an additional current fed from a secondary power source. The secondary current feeding point is located below the MIG contact tip which feeds the primary current. Aoki et al. have clarified the basic characteristics of DCF-MIG.

The feature of the DCF-MIG process is that the welding current is fed at two different points. Previous studies [14,15,16,17] have clarified that the one reason why heat quantity of droplets increase is Joule heating of wire. However, the distance between the feeding point of the primary current and that of the secondary current (herein referred to as “distance of FPs”) was set to a constant value. In this study, the DCF-MIG process was further investigated by focusing on the influence of the distance of FPs.

## 2. Experimental Method

The experiments of the DCF-MIG process with various distances of FPs were carried out. In the welding experiments, the waveforms of welding current and voltage were measured and the arc and droplet transfer were also observed. The heat quantity of droplets were measured by calorimetry. After the welding, the appearance of the weld bead and the macrostructure of the cross section of the weld bead were analyzed.

### 2.1. DCF-MIG Wedling System and Calorimeter

**Figure 1:**
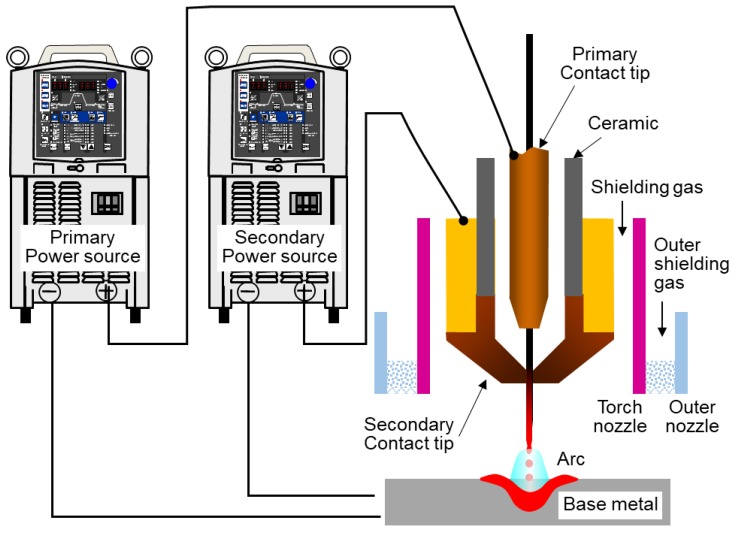
Schematic diagram of DCF-MIGW system.

The structure of the DCF-MIG torch is shown in Figure 1. The basic device configuration was almost the same as that of previous studies [14,15,16,17] except for the distance of FPs to the welding torch. There are two current feeding points which are insulated by ceramic to avoid current conduction. The torch is equipped with a double gas flow structure for the center gas and the shielding gas. The composition of both gas flows is 100% argon (pure Ar). In addition, the outer nozzle was installed around the DCF-MIG torch to feed pure Ar to prevent the bead from oxidation. In the previous study, the distance of FPs was 12 mm. The primary current fed at the primary feeding point has a pulse current and is supplied by a welding power source with constant voltage characteristics, which corresponds to the MIG current in conventional MIG welding. The secondary current fed at the secondary feeding point has a DC waveform and is supplied by a welding power source with constant current characteristics.

**Figure 2:**
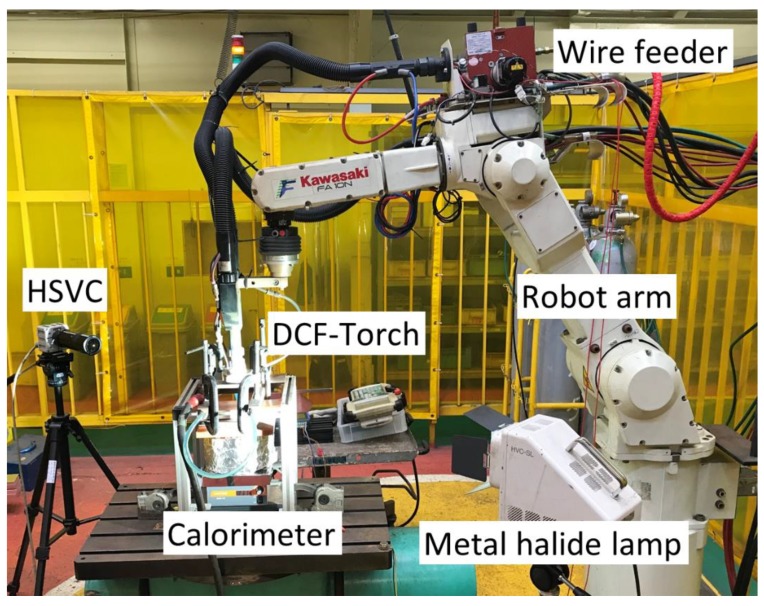
Appearance of the experimental equipment.

The experiment was conducted using a welding robot equipped with a DCF-MIG torch, as shown in Figure 2. The voltage waveforms for the primary and secondary currents were collected by a data logger. The current waveforms were also collected by the data logger through clamp meters. The time resolution of the data collection was 0.1 ms to catch the welding current pulse accurately which has a duration of several milliseconds.

Moreover, the arc shape as well as the growth and detachment processes of droplets were observed using a high speed video camera (HSVC), a telephoto micro lens and a metal halide lamp. The image resolution setting was 1024 × 512 pixels, which corresponds to an image size of 34 × 17 mm. The framerate was 1000 fps, which is sufficient to observe phenomena in the pulse welding. The shutter speed was set to 1/128,000 to reduce the arc brightness. The lamp was placed in line with the optical axis of the HSVC and perpendicular to the welding direction.

**Figure 3:**
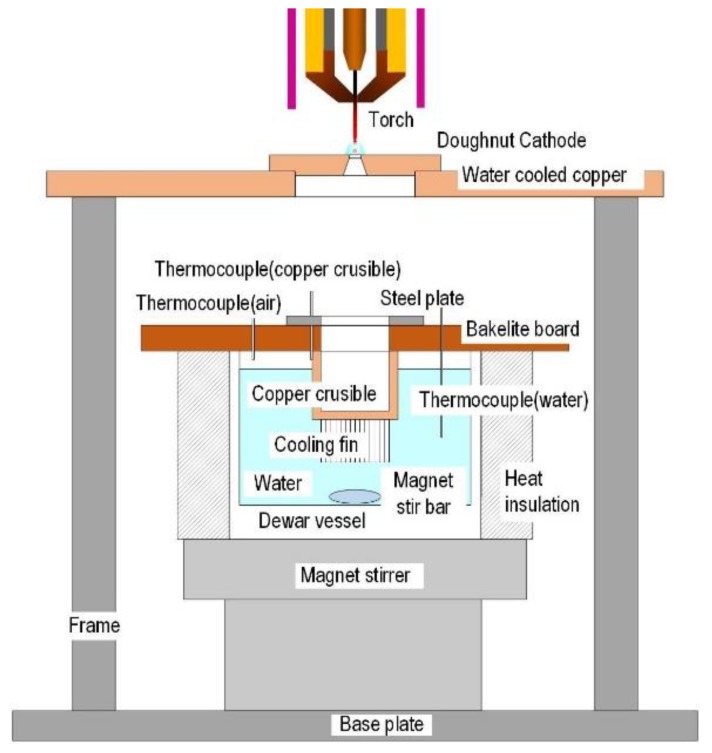
Schematic illustration of a calorimeter.

**Figure 4:**
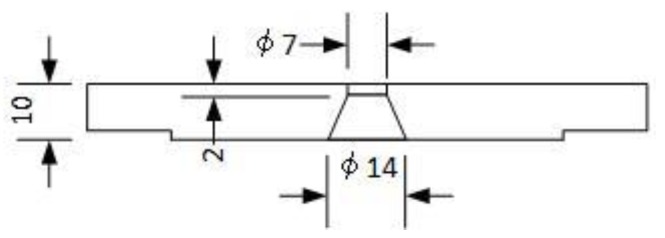
Schematic illustration of a doughnut cathode.

Various types of calorimeters have been reported to measure the temperature or heat quantity of droplets [18,19,20,21]. Figure 3 shows the calorimeter to measure the heat quantity of the droplets in this paper. The calorimeter mainly consists of a dewar vessel, a copper crucible, a doughnut cathode, a magnetic stirrer and thermocouples. The dewar vessel was for the water bath and is covered by heat insulation. The dewar vessel was filled with 1000 g of water. The copper crucible caught the droplets and was steeped in water of dewar vessel. The doughnut cathode (Figure 4) was made of copper and was water-cooled. The doughnut hole allows the passage of the detached droplets into the copper crucible. The water in the dewar vessel was stirred by the magnetic stirrer at 250 rpm. The temperature of water, crucible and atmosphere in the dewar vessel were measured by thermocouples with a sampling time of 50 ms. Then, the collected temperature data were processed using the moving average method to remove noise. The temperature data were collected for more than 180 s from the arc start. For the calculation of heat quantity, temperature data from 120 s to 180 s were used. After 120 s, the temperature of water became almost stable. Welding time was set to 3 s for gathering enough mass of droplets. The mass of gathered droplets was about 2–4 g. The mass of droplets and water were measured by an electric balance with a precision of 10 g. The total amount of the heat quantity of the water, copper crucible and atmosphere in the dewar vessel was divided by the mass of the droplets to determine the heat quantity per unit mass (kJ/g).

### 2.2. Variation of Distance of FPs of DCF-MIG

In order to change the secondary feeding point of the DCF-MIG torch, the secondary contact tips as shown in Table 1 were used. Here, three distances of FPs (12 mm (same as [14,15,16,17]), 16 mm, and 20 mm) were selected. They are referred to as “12 mm Tip”, “16 mm Tip” and “20 mm Tip”. Moreover, conventional MIG welding was carried out in comparison to DCF-MIG welding by using only the primary feeding point by allowing the wire not to touch the secondary tip.

### 2.3. Welding Conditions

A steel solid wire with a diameter of 1.2 mm of JIS Z 3312 G 49 AP 3 M 16 was used. The pure argon gas was used for the center gas, the shielding gas and outer shielding gas. To examine the effect of the position of the secondary feeding point, the distance from the lower edge of the primary contact tip to the work surface (herein referred to as CTWD: contact tip to work distance) was fixed to 30 mm in all the experiments. That is to say that in the case of 12 mm Tip, the distance from the lower edge of the secondary tip to the work surface was 18 mm. In the case of 16 mm Tip, it was 14 mm. In the case of 20 mm Tip, it was 10 mm. In the conventional MIG, the CTWD was set to 30 mm.

The primary power source was set to the “Mild steel pulse MAG welding” mode and wire feeding speed was set to 8 m/min, whose setting current value is 207 A. Concerning the basic pulse shape of the primary power source, the pulse peak current is 403 A, peak period is 1.2 ms and pulse base current is 89 A. The primary power source controls the arc length by pulse frequency modulation. For example, when the arc length is long (e.g., arc voltage is high), the power source stretches the period of the pulse base, which causes a decrease in the average of the pulse current. Then, the wire melting rate becomes low and the arc length shortens.

In the experiment using the calorimeter, the DCF-MIG torch was kept stable. The welding conditions are shown in Table 2. The setting value of the voltage of the primary power source was set in the range from 21 to 27 V to keep the same arc length of about 4 mm in all the experiments.

In order to analyze the bead appearance and the macrostructure of the cross-section, bead-on-plate welding was also conducted. The cross-sectional penetration area below the surface was measured by image processing of the macrostructure. The welding conditions are shown in Table 3. The setting value of voltage of the primary power source was in the range from 30 to 41 V to keep the same arc length of about 4 mm and to prevent a short circuit transfer in all the experiments. Under the same arc length, the setting voltage value was set to be larger as the distance of FPs was longer and the secondary current was smaller. The setting current of the secondary power source was in the range from 25 to 100 A. The base metal was mild steel (JIS SS400) with a 9 mm thickness and its surface scale was removed by a grinder.

## 3. Experimental Results

### 3.1. Mesurement of Waveforms of Current and Voltage

**Figure 5:**
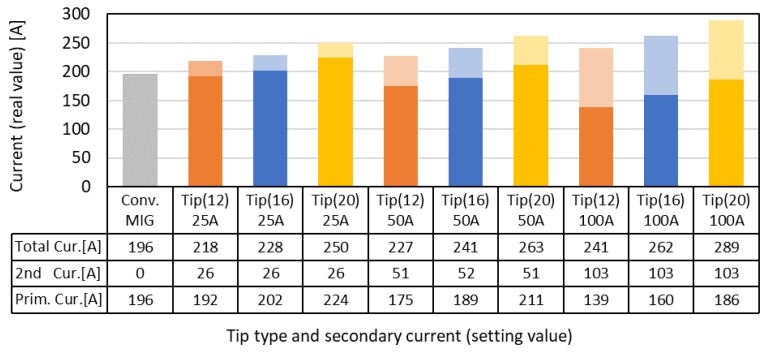
Average value of the current (lower: primary current (Prim. Cur.), upper: secondary current (2nd Cur.)).

Figure 5 shows the average values of the total current (“Total Cur.”), which is the sum of the primary current (“1st Cur.”) and the secondary current as a function of the secondary current. This data was measured with a calorimetric experiment at the same time. The primary current decreased and the total current increased as the secondary current increased. Concerning the distance of FPs, the total current was found to increase with increase of the distance of FPs under the same secondary current. Furthermore, the total current of DCF-MIG was always larger than that of the conventional MIG.

**Figure 6:**
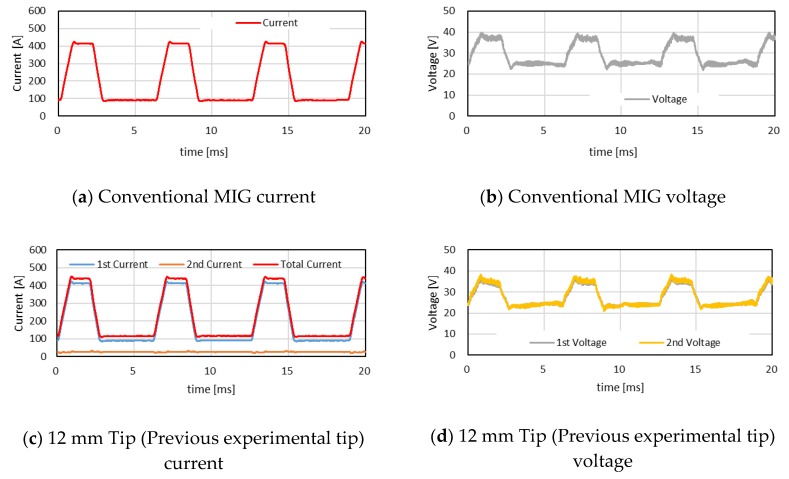

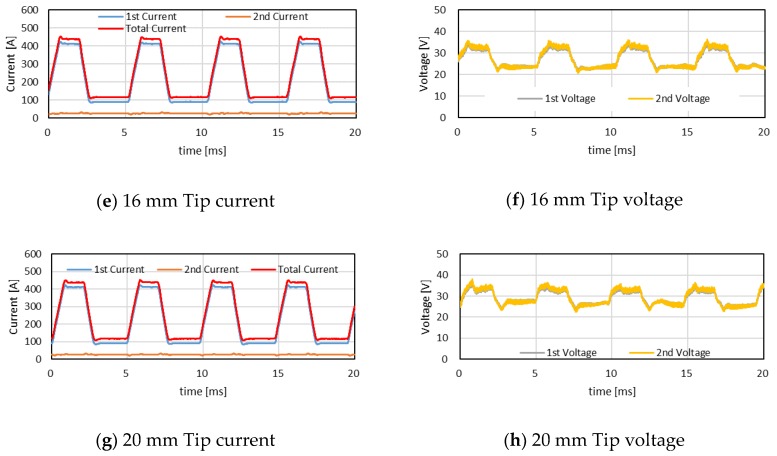
Waveforms of welding current and voltage.

An example of the waveform of the current and voltage under the secondary current of 25 A is shown in Figure 6. The voltages of the primary power source were set to 26–29 V to keep the constant arc length of 4 mm. The measured average voltages were about 27–29 V at any distance of FPs. Concerning the current waveform, it was found that the pulse period became shorter as the distance of FPs increased. The short pulse period means an increase of the average value of the primary current as well as an increase of the total current.

### 3.2. Observation of Droplets

Table 4 shows the images of droplet transfer taken by HSVC at 1000 fps in the secondary current of 50 A and 100 A. For want of space, the secondary current of 25 A, whose droplet transfer was similar to that of 50 A, was omitted. Likewise, since the droplet transfer of 16 mm Tip was similar to that of 12 mm Tip, their images were also omitted. The series of images shows the results in the period from the pulse peak to the next pulse peak. One image corresponds to 1 ms period. An increase of the pulse period caused by the primary current decrease was observed. When the secondary current was 25 A or 50 A, “1 pulse 1 drop” transfer was realized in any distance of FPs. But when the secondary current was 100 A, “1 pulse multi drops” transfer occurred, leading to arc instability, except for the case of 20 mm Tip. The reason for this multi drops is that the wire melting rate was increased by the high total current as well as the long pulse base period. The multi drops easily cause a short circuit transfer, which brings instability of arc control by the welding power source. One method to avoid this is to enlarge the arc length, e.g., setting a high voltage. But setting a high voltage tends to melt the contact tip, so this is the constraint of using a long distance of FPs.

### 3.3. Analysis of Apperance and Cross Section of Bead

Table 5 shows the appearance of the bead as well as the macrostructure of the cross section of the bead in the case of a secondary current of 25 A. The measured average current, voltage and flank angles are also presented. It was found that the width of the bead became larger as the distance of FPs increased. Concerning the macrostructure, the flank angle also increased as the distance of FPs increased. As a result, the bead shape seemed to be clearly improved in the case of larger distance of FPs The microstructure of DCF-MIG with 20 mm Tip is also shown in Table 5. A coarse structure similar to that in [5] is seen. This structure appears in the weld metal with a low oxygen content.

**Figure 7:**
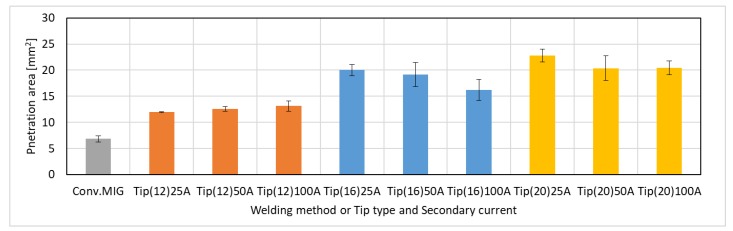
Penetration area of beads.

Figure 7 shows the average of the cross-sectional penetration area below the surface obtained from four beads in each welding condition. The area increased as the distance of FPs increased. Consequently, it was found that the DCF-MIG was able to obtain a larger penetration area by adding only a small secondary current of around 25 A in comparison with the conventional MIG, and the penetration area significantly increased as the distance of FPs increased.

### 3.4. Measurement of Heat Quantity of Droplet

**Figure 8:**
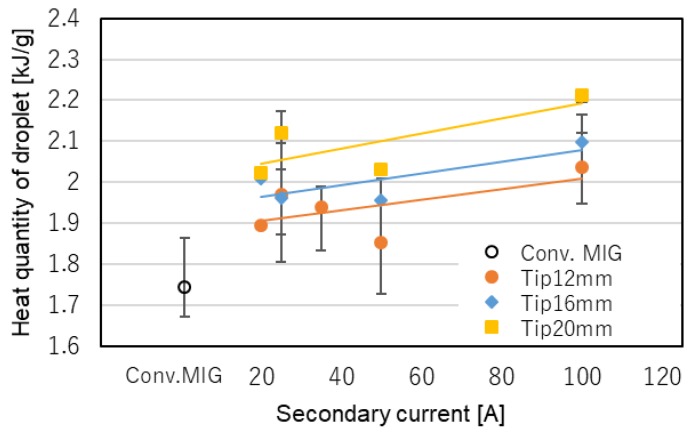
Result of quantity of heat of droplet measurement.

Figure 8 shows the results of the measurement of the heat quantity of the droplets. It is clear that the heat quantity of the droplets of DCF-MIG is larger than that of the conventional MIG. In the case of DCF-MIG, the heat quantity of droplets increased as the distance of FPs and the secondary current increased. These tendencies can be explained by the increase of the total current.

These results show the major reason for the improvement of bead shape and large penetration.

## 4. Discussion

From the above experimental results, it is clarified that the DCF-MIG was able to increase the total current and the heat quantity of droplets by feeding the secondary current. Moreover, the effect of the distance of FPs was found to be especially significant (i.e., the heat input increased as the distance of FPs increased). In this section, the energy balance of DCF-MIG wire is discussed through a simplified calculation.

The relationship between wire melting speed *V_m_* [mm/s] and the heat quantity of droplet *H*_0_ [J/mm^3^] is represented by the equation of Halmoy [22].

(1) $$V_{m}=\frac{1}{H_{0}+b}\left(\phi j+aLj^{2}\right),$$

where *ϕ* [V] is the equivalent voltage of melting anode by arc, *j* [A/mm^2^] is the current density, *L* [mm] is the wire extension, *a* [Ω·mm] is the constant of specific resistance of wire, and *b* [J/mm^3^] is the constant, depending on the specific resistance. Equation (2) is obtained by solving Equation (1) for *H*_0_:

(2) $$H_{0}=\frac{\phi j}{V_{m}}+\left(\frac{aLj^{2}}{V_{m}}-b\right).$$

Here, the first term on the right side of Equation (2) indicates the heat input from the arc. The second term indicates Joule heating, which is the first order approximation of experimental data from the four-terminal sensing. Based on the experimental research by Maruo et al. [23], the constants of Equation (2) are determined as follows: ϕ is 5.7 V, *a* is 9.40 × 10^−4^ Ω·mm at 1000 K and b is 1.78 J/mm^3^.

**Figure 9:**
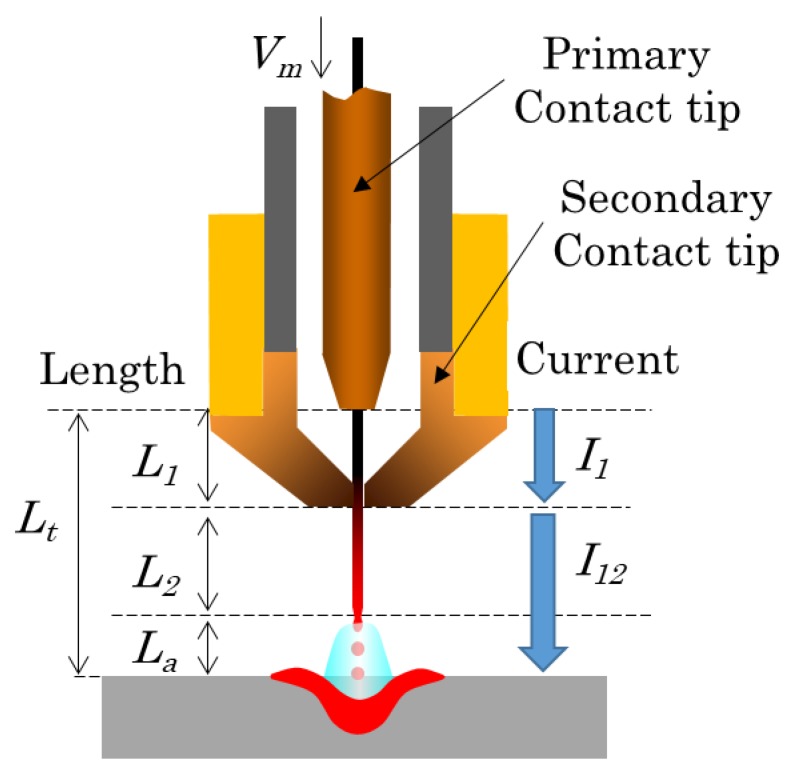
Schematic representation of the DCF-MIGW torch.

The torch structure of DCF-MIG is illustrated in Figure 9 [15]. The primary current *I_1_* flows through the section from the primary feeding point to the secondary feeding point. Through the section from the secondary feeding point to the tip of the wire, the total current *I_12_*, which is the sum of the primary current *I_1_* and the secondary current *I_2_* flows. Here, currents *I_1_* and *I_12_* are divided by a wire cross-sectional area to calculate current density *j_1_* and *j_12_*. Then, Equation (3) is derived by expanding Equation (2). The term of Joule heating on the section between the feeding points is added as follows:

(3) $$H_{0}=\frac{\phi j_{12}}{V_{m}}+\left(\frac{aL_{1}j_{1}^{2}}{V_{m}}-b\right)+\left(\frac{aL_{2}j_{12}^{2}}{V_{m}}-b\right).$$

Furthermore, in the DCF-MIG, the primary current decreased by several tens of amperes by feeding the secondary current. This caused the increase of the pulse period by the pulse frequency modulation controller of the primary power source. Concerning Joule heating calculation, it is considered that using an effective current value is more suitable than using the average value of the pulse current [24]. The effective current value is defined to be equal to the value of the direct current that would produce the same average power dissipation in a resistive load. The effective current value of a non-sine wave such as the welding pulse current is represented ass

(4) $$I_{e}=\sqrt{\frac{1}{T}\int_{0}^{T}i{\left(t\right)}^{2}dt}.$$

where *I_e_* represents the effective value, *i(t)* indicates the instantaneous value of the pulse current and *T* is the pulse period. Considering Equation (4), since pulse peak waveform does not change in the pulse frequency modulation, the increase of the pulse period leads to a decrease of the effective value. For example, in the case of 12 mm Tip, when the secondary current was 25 A and the pulse period was about 6.5 ms, the averaged primary current was 192 A and the effective value of the primary current became 212 A. When the secondary current was 100 A and the pulse period was about 12.5 ms, the averaged primary current was 141 A and the effective current value was 164 A. The effective current value was mostly larger than the averaged current value.

**Figure 10:**
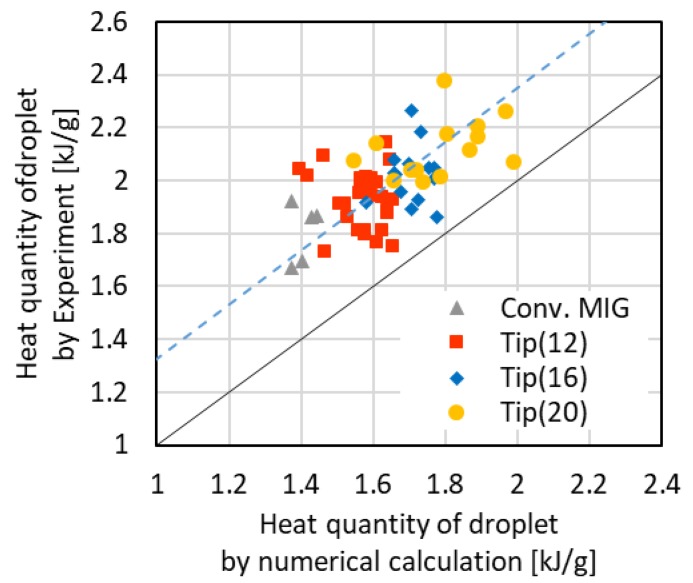
Heat quantity of a droplet by numerical calculation and experimental value.

Figure 10 shows the relationship between the heat quantity obtained from the calculation by Equation (3) using the effective current value and the measured heat quantity. The effective current value of this calculation was determined from the experiment. The dotted line in the graph is the first approximation of data. The first approximation line runs approximately parallel to the line, which is a 1 to 1 relationship between the calculated heat quantity and the measured heat quantity. These calculation results indicate that the simplified calculation roughly expresses the measured tendency.

**Figure 11:**
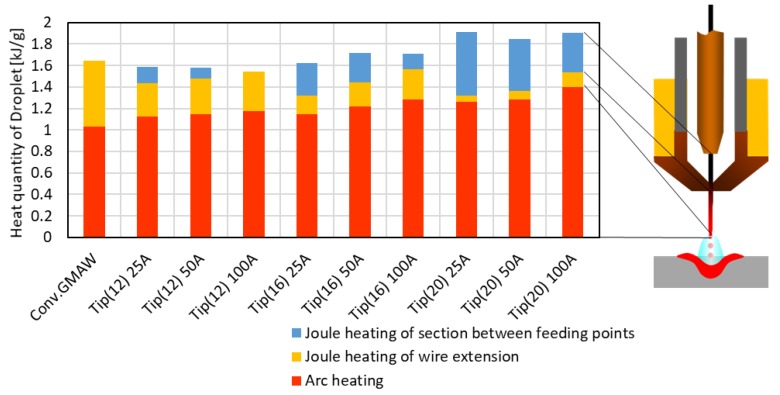
Contribution of each section to the quantity of the heat of a droplet by numerical calculation.

Figure 11 shows the details of the calculation results on the influence of the distance of FPs and the secondary current, obtained using Equation (3) with an effective current value. The upper part of bar graph represents Joule heating between the primary feeding point and the secondary feeding point, the middle part of bar graph represents Joule heating in wire extension, and the lower part of bar graph represents the arc heating.

It was found that the contribution of each section to the quantity of heat of droplet changes with the secondary current and/or distance of FPs. For example, as the distance of FPs increases, the Joule heating between feeding points naturally increases. In contradiction to this, Joule heating at wire extension decreased because the wire extension became short. Then, concerning the change of the secondary current, as the secondary current increased, Joule heating between the feeding points decreased and Joule heating in the wire extension increased. This phenomenon is caused by the primary current decreasing as the secondary current increases. The arc heating increases as the total current increases.

In the above calculation, the mechanism to cause the increase in heat quantity with the increase of the distance of FPs is presented. However, it is not enough explained that the heat quantity increases with the secondary current. One of the reasons for this inconsistency is the use of the simplified calculation of Halmoy. In order to discuss this point more in detail, a numerical simulation is planned to be carried out.

Next, influence of a rise in heat input using DCF-MIG is discussed. In the bead on plate welding experiment, the heat input of conventional MIG was about 1.4 kJ/mm and that of DCF-MIG with a secondary current of 100 A and 20 mm Tip corresponding to the highest total current condition was 2.68 kJ/mm. Typically, a maximum heat input for TMCP (Thermo-Mechanically Controlled Processing) steel of 15 mm thickness is 2.5 kJ/mm [25]. If the heat input exceeds the restriction value, problems such as decrease in toughness of weld metal and HAZ softening occur [26,27]. In this study, the work piece with a thickness of 9 mm was used, but a final target of this study is a medium and heavy thickness plate (much thicker). Furthermore, DCF-MIG was able to flexibly control the heat input from the low value corresponding to conventional MIG up to the maximum heat input restriction of TMCP steel. This controllability is the advantage of DCF-MIG.

However, the excessive heat input by DCF-MIG is considered to cause a formation of coarse grain to decrease the toughness or other problems related to a high heat input. In the case of conventional MIG at a wire feeding speed of 8 m/min, the total heat input to the work piece is estimated to be 4.80 kJ/s, which is calculated by the measured current of 197 A, a measured voltage of 29.0 V and a heat efficiency of 0.84 [28]. The heat quantity of the droplet transferred to the work piece per second was 2.11 kJ/s. Accordingly, the heat quantity of droplet accounts for 44.1% of the total heat input to the work piece in a conventional MIG process. This ratio is suitable for the value indicated by DuPont [28]. On the other hand, in DCF-MIG with 20 mm Tip and a secondary current of 100 A, the total heat input to the work piece is estimated to be 7.14 kJ/s, which is calculated by measuring the total current of 291 A, a measured voltage of 29.2 V and a heat efficiency of 0.84. The heat quantity of droplet per second was 2.64 kJ/s. As a result, the ratio of heat input by droplet to total heat input was 37.0%. This value means that the heat input by the arc plasma to the work piece in DCF-MIG is larger than that of conventional MIG. This extra heat input by the arc plasma is considered to contribute preheating a wide range of work piece surface to improve the wettability in the DCF-MIG process. However, it is thought that there is only a slight influence of extra heat input on welding metal microstructure because almost the same microstructure with that of [5] was obtained, as presented in Figure 8.

The previous DCF-MIG studies focused on the influence of the electrical conductivity of the wire material [15,16]. These studies clarified that the increase in the droplet temperature in DCF-MIG was significant in the case of a wire material with a low electrical conductivity. In the present study, this effect was suggested to be further improved by optimizing the welding torch structure. In the case of welding of a LNG tank, a nickel-based alloy wire is generally used. The nickel-based alloy has a lower electrical conductivity than that of the steel used here, so the welding is expected to be significantly improved by using DCF-MIG. As a next step, DCF-MIG is planned to be applied to a practical use by employing the wire material especially with a low electrical conductivity.

## 5. Conclusions

A MIG welding process was developed, called duplex current feeding MIG (DCF-MIG). The effect of the distance of the current feeding points of DCF-MIG was clarified by experiments. The conclusions are summarized as follows:

1. The droplet heat quantity was found to significantly increase with the increase of the distance of the feeding points.
2. The increase of the droplet heat quantity by DCF-MIG strongly contributed to improve the bead shape and penetration.
3. The droplet heat quantity calculation using effective current value of DCF-MIG was derived from the simplified calculation and the results roughly agree with the experimental data.

## Figures and Tables

**Table 1:** Secondary contact tips for experiments.

	12 mm Tip (Previous Experiment Tip)	16 mm Tip	20 mm Tip
Distance Between Primary Contact Tip and Secondary Contact Tip	12 mm	16 mm	20 mm
Appearance	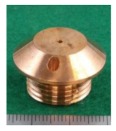	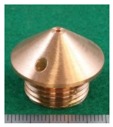	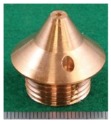

**Table 2:** Welding conditions for calorimetric experiment.

Welding Process	Wire Feeding Speed	Primary Power Source Setting Voltage	Secondary Setting Current
Conventional MIG	8 m/min	26 V	-
DCF-MIG	8 m/min	21~27 V	20 A, 25 A 50 A, 100 A

**Table 3:** Welding conditions for bead on the plate welding experiment.

Welding Process	Welding Speed	Wire Feeding Speed	Primary Power Source Setting Voltage	Secondary Setting Current
Conventional MIG	30 cm/min	8 m/min	31 V	-
DCF-MIG	30 cm/min	8 m/min	30~41 V	25 A 50 A, 100 A

**Table 4:** Droplet images taken by a high speed video camera.

Welding Cond.		Image of Droplet 1 Frame = 1 ms
Conv. MIG		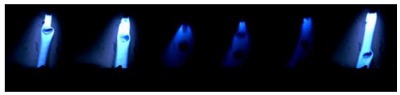
2ndCur.	Tip	
50 A	Tip 12 mm	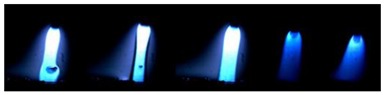 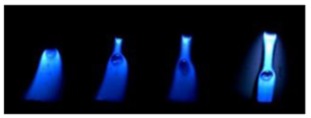
	Tip 20 mm	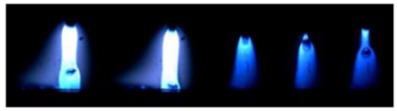 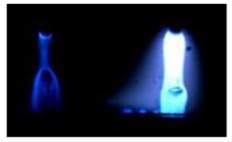
100 A	Tip 12 mm	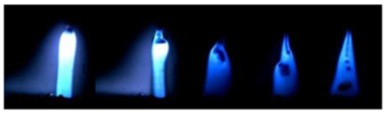 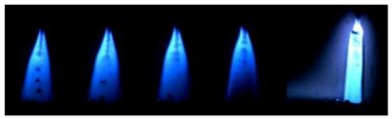
	Tip 20 mm	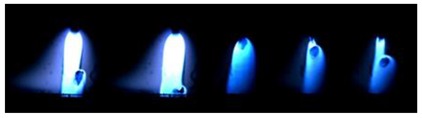 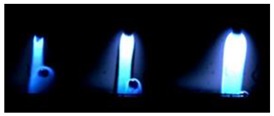

**Table 5:** Appearance and cross section of weld beads of conventional MIGW and DCF-MIGW with a second current of 25 A as well as the microstructure of DCF-MIG in 20 mm Tip with a second current of 100 A.

Conventional MIG	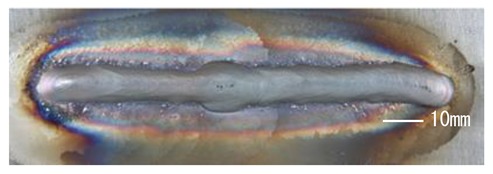	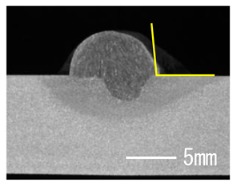
	Cur. = 194 A, Vol. = 36.6 V	Flank ang. = 95.4 (ave.)
DCF-MIG 12 mm Tip	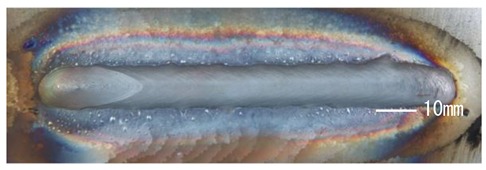	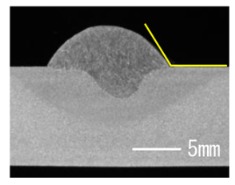
	Total cur. = 258 A, Vol. = 35.3 V	Flank ang. = 117 (ave.)
DCF-MIG 16 mm Tip	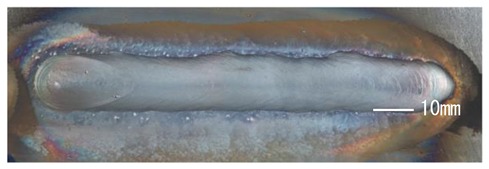	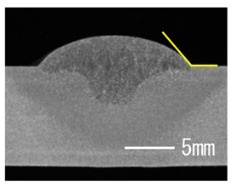
	Total cur. = 283 A, Vol.=35.7 V	Flank ang. = 134 (ave.)
DCF-MIG 20 mm Tip	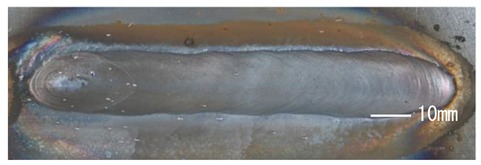	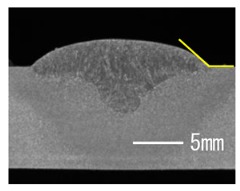
	Total cur. = 306 A, Vol. = 36.7 V	Flank ang. = 140 (ave.)
Microstructure DCF-MIG 20 mm Tip 2nd Cur 100 A